# Coronary Artery Aneurysm or Ectasia as a Form of Coronary Artery Remodeling: Etiology, Pathogenesis, Diagnostics, Complications, and Treatment

**DOI:** 10.3390/biomedicines12091984

**Published:** 2024-09-02

**Authors:** Patrycja Woźniak, Sylwia Iwańczyk, Maciej Błaszyk, Konrad Stępień, Maciej Lesiak, Tatiana Mularek-Kubzdela, Aleksander Araszkiewicz

**Affiliations:** 1 1st Department of Cardiology, Poznan University of Medical Sciences, Długa 1/2 Street, 61-848 Poznań, Poland; 2 Department of Radiology, Poznan University of Medical Sciences, 61-701 Poznań, Poland; 3 Department of Coronary Artery Disease and Heart Failure, Jagiellonian University Medical College, Prądnicka 80 Street, 31-202 Kraków, Poland

**Keywords:** coronary artery aneurysm, coronary artery ectasia, atherosclerosis, coronary artery disease

## Abstract

Coronary artery aneurysm or ectasia (CAAE) is a term that includes both coronary artery ectasia (CAE) and coronary artery aneurysm (CAA), despite distinct phenotypes and definitions. This anomaly can be found in 0.15–5.3% of coronary angiography. CAE is a diffuse dilatation of the coronary artery at least 1.5 times wider than the diameter of the normal coronary artery in a patient with a length of over 20 mm or greater than one-third of the vessel. CAE can be further subdivided into diffuse and focal dilations by the number and the length of the dilated vessels. Histologically, it presents with extensive destruction of musculoelastic elements, marked degradation of collagen and elastic fibers, and disruption of the elastic lamina. Conversely, CAA is a focal lesion manifesting as focal dilatation, which can be fusiform (if the longitudinal diameter is greater than the transverse) or saccular (if the longitudinal diameter is smaller than the transverse). Giant CAA is defined as a 4-fold enlargement of the vessel diameter and is observed in only 0.02% of patients after coronary. An aneurysmal lesion can be either single or multiple. It can be either a congenital or acquired phenomenon. The pathophysiological mechanisms responsible for the formation of CAAE are not well understood. Atherosclerosis is the most common etiology of CAAE in adults, while Kawasaki disease is the most common in children. Other etiological factors include systemic connective tissue diseases, infectious diseases, vasculitis, congenital anomalies, genetic factors, and idiopathic CAA. Invasive assessment of CAAE is based on coronary angiography. Coronary computed tomography (CT) is a noninvasive method that enables accurate evaluation of aneurysm size and location. The most common complications are coronary spasm, local thrombosis, distal embolization, coronary artery rupture, and compression of adjacent structures by giant coronary aneurysms. The approach to each patient with CAAE should depend on the severity of symptoms, anatomical structure, size, and location of the aneurysm. Treatment methods should be carefully considered to avoid possible complications of CAAE. Simultaneously, we should not unnecessarily expose the patient to the risk of intervention or surgical treatment. Patients can be offered conservative or invasive treatment. However, there are still numerous controversies and ambiguities regarding the etiology, prognosis, and treatment of patients with coronary artery aneurysms. This study summarizes the current knowledge about this disease’s etiology, pathogenesis, and management.

## 1. Introduction

Coronary artery aneurysm or ectasia (CAAE) is a term that includes both coronary artery aneurysm (CAA) and coronary artery ectasia (CAE), despite distinct phenotypes and definitions. This anomaly can be found in 0.15–5.3% of coronary angiography [1,2,3,4,5,6] and is more frequent in men than in women (83% vs. 60%) [7,8]. However, the development and prevalence of coronary angiography, multislice computed tomography, and magnetic resonance imaging have increased the detection of coronary aneurysms. Although CAAE may be asymptomatic and diagnosed incidentally, some symptomatic patients may manifest complications of unstable angina, acute myocardial infarction (MI), arrhythmias, or sudden cardiac death. Major adverse cardiovascular events (MACEs), defined as the composite of cardiovascular mortality, myocardial infarction (MI), stroke, and revascularization, occur in up to 10% of CAAE patients per year [9,10].

Atherosclerosis is the most common etiology of CAAE. Some scientists have estimated that 50% of CAE or aneurysms are related to atherosclerosis, while 20–30% of cases may be classified as congenital anomalies [11,12]. Other etiological factors include Kawasaki disease, systemic connective tissue diseases, infectious diseases, vasculitis, congenital anomalies, genetic factors, and idiopathic CAA [6,13]. Although some pathophysiological and clinical risk factors for the development of CAAE have been identified, detailed mechanisms are not yet known [3,14]. Moreover, so far, the data on patients with CAAE include case reports, case series, and registries (the International Coronary Artery Aneurysm Registry [CAAR] and Coronary Artery Ectasia Database—Poland [CARED-POL]) [3,14,15,16,17,18,19,20,21].

However, there are still numerous controversies and ambiguities regarding the etiology, prognosis, and treatment of patients with coronary artery aneurysms. This study summarizes the current knowledge about this disease’s etiology, pathogenesis, and management. 

## 2. Definition and Clinical Presentation

**Figure 1:**
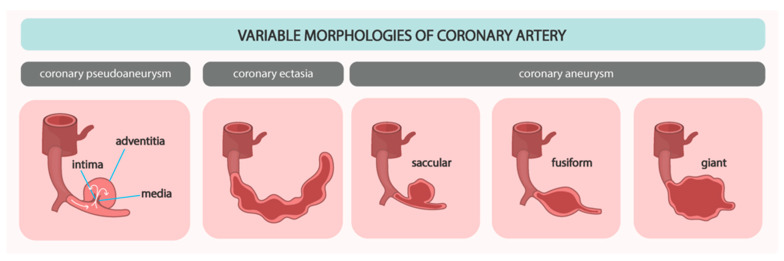
Possible morphologies and clinical manifestations of aneurysmal dilatation of coronary arteries. White arrow indicates the blood flow.

Coronary artery aneurysm or ectasia (CAAE) is a term that includes both coronary artery aneurysm (CAA) and coronary artery ectasia (CAE), despite distinct phenotypes and definitions [1,2]. CAE is defined as a diffuse dilatation of the coronary artery at least 1.5 times wider than the diameter of the normal coronary artery in a patient with a length of over 20 mm or greater than one-third of the vessel. CAE can be further subdivided into diffuse and focal dilations by the number and the length of the dilated vessels. Histologically, it presents with extensive destruction of musculoelastic elements, marked degradation of collagen and elastic fibers, and disruption of the elastic lamina. Conversely, CAA is a focal lesion manifesting as a focal dilatation, which can be saccular (if the transverse diameter is greater than the longitudinal) or fusiform (if the longitudinal diameter is greater than the transverse) (Figure 1) [14]. Markis et al. have provided a classification of CAE, including four types (Table 1) [3]. Giant CAA is defined as a 4-fold enlargement of the vessel diameter and is observed in only 0.02% of patients after coronary. An aneurysmal lesion can be either single or multiple. It can be either a congenital or acquired phenomenon.

Aneurysms are most often located in the proximal and middle segments of the right coronary artery (40–73%), the proximal segment of the left anterior descending artery (10–51%), and less often in the left circumflex coronary artery (4–43%). Occasionally (2–9%), they appear in the left main coronary artery. 

Subclassification of CAAE based on morphological or intravascular imaging factors is presented in Table 1 and Table 2.

## 3. Etiology and Pathogenesis

The exact pathogenesis of CAAE is not well established. In 1987, Glagov described the phenomenon of compensatory dilation of the arterial lumen at the site of early atherosclerotic lesions, compensating for the accumulation of atherosclerotic plaques [22]. This phenomenon was called positive remodeling of the coronary arteries [23]. However, not all patients with atherosclerotic lesions develop local dilatation of coronary arteries. 

The scientists identified several clinical and pathophysiological factors that probably impact aneurysm formation. These include hypertension, smoking, male sex, hyperhomocysteinemia, and atherosclerotic processes triggered and accelerated by Epstein–Barr virus infection. The genetic factors include the presence of *HLA-DR B1813*, *DR16*, *DQ2*, and *DQ5* alleles, occurrence of the *5A* allele of the *MMP-3* gene, and deletion–insertion polymorphism of the *ACE* gene (*ACE DD* genotype) [1,14,24,25,26,27]. 

The inflammation in the dilated segment can be a causative factor in the development of aneurysms in atherosclerotic vessels. However, it can also be an independent process in the vessel wall.

Researchers have found that the occurrence of coronary aneurysms is associated with an intensified inflammatory process involving all layers of the vessel. Inflammation in the dilated segment increases the risk of plaque rupture [23]. Patients with isolated coronary aneurysms have higher serum levels of C-reactive protein and interleukin 6 compared to patients with obstructive coronary artery disease and without ischemic heart disease [28,29]. Increased plasma levels of soluble adhesive molecules, such as VCAM-1, ICAM-1, and E-selectin, as well as increased activity of matrix metalloproteinases (MMPs), are also an expression of the increased inflammatory process in patients with CAAE [14,29,30].

Endocan, also known as endothelial cell-specific molecule-1 (ESM-1), is a soluble proteoglycan that is mainly secreted by endothelial cells in response to inflammatory cytokines. Endocan regulates the functioning of pathophysiological processes, including inflammation, angiogenesis, and cell adhesion [31,32]. Endocan plasma levels can increase in response to endothelial activation and dysfunction; hence, it is a potential immuno-inflammatory marker related to cardiovascular disease [33]. Endocan is a potential marker of damage to the vascular wall due to inflammation in the course of atherosclerosis. Furthermore, endocan can be regarded as a marker of vascular remodeling due to the imbalance between pro- and anti-angiogenic processes. The endocan level reflects the intensity of the above processes. Therefore, it correlates with the severity of CAE, measured as the total volume of dilation [34].

Another potential mechanism responsible for the formation of CAAE is genetic determinism. In another study, the authors performed analysis using ClinVar method. They revealed that all patients had single-nucleotide variants (SNVs) in several genes: *ADAMTS13*, *GALC*, *ITPKB*, *NPC1*, and *PERM1*. *ADAMTS13*, *ITPKB*, and *PERM1* had missense mutations, while *GALC* and *NPC1* had mutations in splicing sites [13].

Metalloproteinases are enzymes that decompose structural proteins of connective tissue (matrix). Degradation of the extracellular matrix weakens elastic and muscle tissue in the lamina elastica, leading to weakening and thinning of the vessel wall. As a result of mechanical forces affecting the weakened wall, an aneurysm is formed. 

Atherosclerotic plaques and macrophages are the main sources of MMP-9. The level of MMP-9 is directly correlated with the activity of fibrinogen, C-reactive protein, and IL-6, which are markers for predicting the risk of myocardial infarction (MI) [35]. Research has found that neutrophils are a source of MMP-9 [36]. Furthermore, MMP-9 was found to participate in post-MI ventricular remodeling through impaired angiogenesis and tissue remodeling [37]. Nevertheless, the MMP-9 deletion seems to stimulate neovascularization in remodeling the myocardium, rather than decreasing it [38]. Blastocysts are a direct source of MMP-2 [39].

Thanks to the prostaglandin-dependent mechanism E2-cAMP, the regulation of MMP gene expression is carried out at the transcriptional level. Several inflammatory cytokines, hormones, and growth factors can regulate transcriptional activity. The activity of MMPs is regulated by tissue-specific inhibitors of metalloproteinases (TIMP-1, -2, -3, and TIMP-4), which are secreted by myocytes and macrophages. The activity of MMPs is increased by growth factors and decreased by various interleukins. Iwańczyk et al. demonstrated that the plasma levels of MMP-8 were significantly higher in the CAAE group than in the coronary artery disease (CAD) and control groups [40]. Another study presented elevated levels of MMP-2, MMP-3, MMP-9, and MMP-12, as well as decreased concentrations of TIMP, in the vascular walls of coronary aneurysms [41]. They also found that plasmin strongly stimulates the conversion of pro-MMP to the active form. In patients with CAE, plasminogen activator inhibitor-1 (PAI-1) activity is significantly decreased, indirectly increasing the activity of MMPs [42]. The connection between decreased PAI-1 and increased plasmin activity is as follows: the lower levels of active PAI-1 (as opposed to latent PAI-1) will lead to increased activity of tPA and uPA, which are the active proteases that convert the inactive zymogen plasminogen to active plasmin. Hence, this may lead to increased MMP activity. 

Therefore, the direct cause of aneurysms is an intensified inflammatory process, which causes the imbalance between proteolytic enzymes and their inhibitors in the vessel wall, especially in the elastic membrane [14,41]. 

Senzaki et al. observed a vital role in the inflammatory process and activation of MMPs in patients with non-atherosclerotic aneurysm causes, including Kawasaki disease [43]. The pathogenesis of aneurysm complications also includes the tendency to form blood clots within the lumen. In addition to the slow blood flow and the instability of atherosclerotic plaques within the aneurysm (intensification of the local inflammatory process), platelet hyperactivity also plays an important role [44]. Histological examination of coronary aneurysms revealed hyalinization, lipid deposition, intimal disruption, calcifications, local fibrosis, and intravascular bleeding.

Studies on the role of miRNA in patients with CAAE are scarce. The researchers analyzed the differences in the expression of previously preselected miRNAs as potential markers of CAAE. They found that miR-451a and miR-328 significantly improved the CAAE prediction. They concluded that miR-451a and miR-328-3p are significant markers of CAAE compared to patients with CAD and control individuals [45,46].

Another potential cause may be immunoglobulin G4-related disease (IgG4-RD). IgG4-RD is a systemic, chronic disease characterized by inflammatory fibrosis and high serum IgG4 levels. In the course of the disease, IgG4-positive plasma cells infiltrate target organs and may involve the pancreas, lacrimal glands, biliary tract, thyroid, salivary glands, orbits, lymph nodes, kidneys, or retroperitoneum. Furthermore, IgG4-RD may manifest as vasculitis involving large to medium-sized vessels such as the aorta or the common iliac, carotid, and coronary arteries. Yadav et al. described a case of a 55-year-old male patient with a giant CAA involving the left anterior descending artery and multiple visceral and intercostal artery aneurysms found on CT angiography, secondary to IgG4 vasculopathy [47].

All patients with symptomatic, active IgG4-RD require treatment, some urgently, according to the International Consensus Guidance published in 2015 [48]. Glucocorticoids are the first-line agents. However, at least one-third of patients experience relapse after discontinuation of steroids. The relapse is an indication to consider steroid-sparing agents [49]. Despite the infectious complications, rituximab (RTX), an anti-CD20 monoclonal antibody, represents a promising strategy. Among patients without therapy, improvement occurred in 43% of cases [49]. Nonetheless, we should remember the controversial possibility of corticosteroids causing accelerated aneurysmal degeneration and increased rupture risk [50,51,52].

## 4. Diagnostics

**Figure 2:**
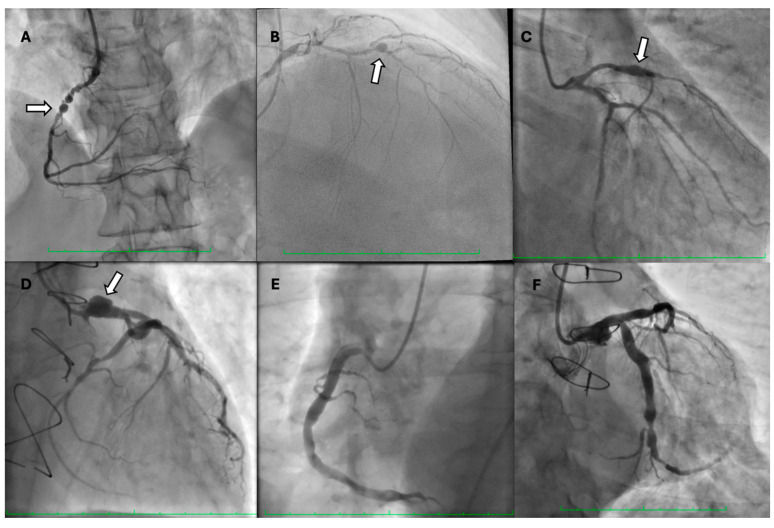
Coronary angiograms showing (**A**) saccular aneurysm of the right coronary artery (arrow), (**B**) saccular aneurysm of the mid-segment of the left anterior descending artery (arrow), (**C**) fusiform aneurysm of the left anterior descending artery (arrow), (**D**) saccular aneurysm of the left main coronary artery (arrow), (**E**) coronary artery ectasia of the right coronary artery, and (**F**) coronary artery ectasia of the left circumflex artery.

**Figure 3:**
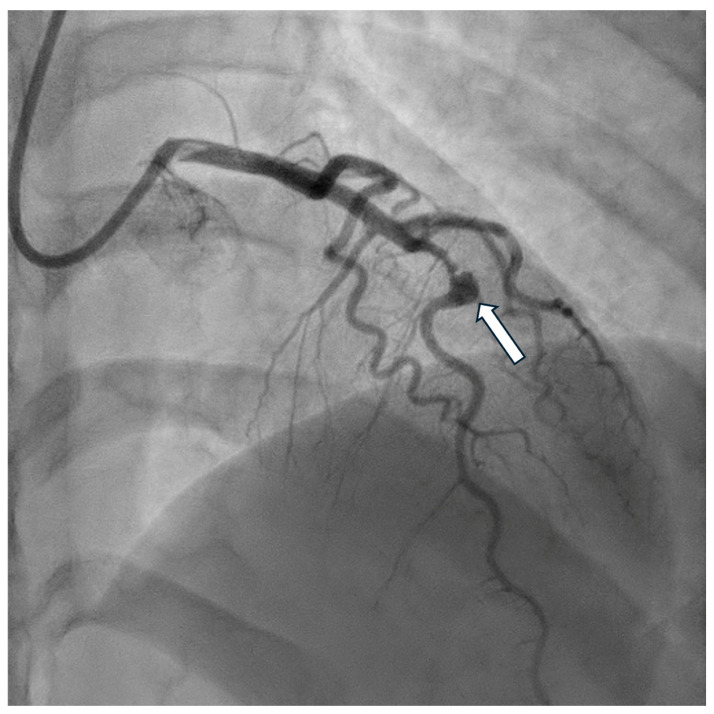
Coronary angiography of the saccular aneurysm (arrow) in the mid-segment of the left anterior descending artery.

**Figure 4:**
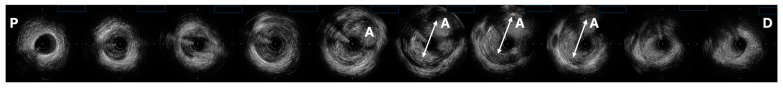
IVUS of the left anterior descending artery before the aneurysmal lesion, within and after the aneurysmal lesion. We have given many cross-sections to present the proximal and distal vessel references and aneurysm-to-reference ratio, showing how large the lesion is. We have also provided the legend to make it easily readable: A—aneurysm; P—proximal; D—distal; arrows—maximal vessel diameter of aneurysm.

Invasive assessment of CAAE is based on coronary angiography (Figure 2). Nevertheless, this technique has limitations, as the actual size of the aneurysm may be underestimated in the presence of a thrombus. Furthermore, the turbulent flow in the dilated coronary segment can impair optimal imaging during coronary angiography [53]. Suboptimal imaging of the aneurysm hinders a thorough evaluation, including the presence of a thrombus. In such cases, intravascular ultrasound (IVUS) can be conclusive. Intracoronary imaging is a helpful tool for assessment of the accurate size of CAA, distinguishing between true aneurysms, pseudoaneurysms, and segments imitating aneurysms due to plaque rupture or adjacent stenosis, as it provides more precise visualization of vessel wall structures (Figure 3 and Figure 4). IVUS also allows proper stent sizing if percutaneous coronary intervention (PCI) is necessary. 

**Figure 5:**
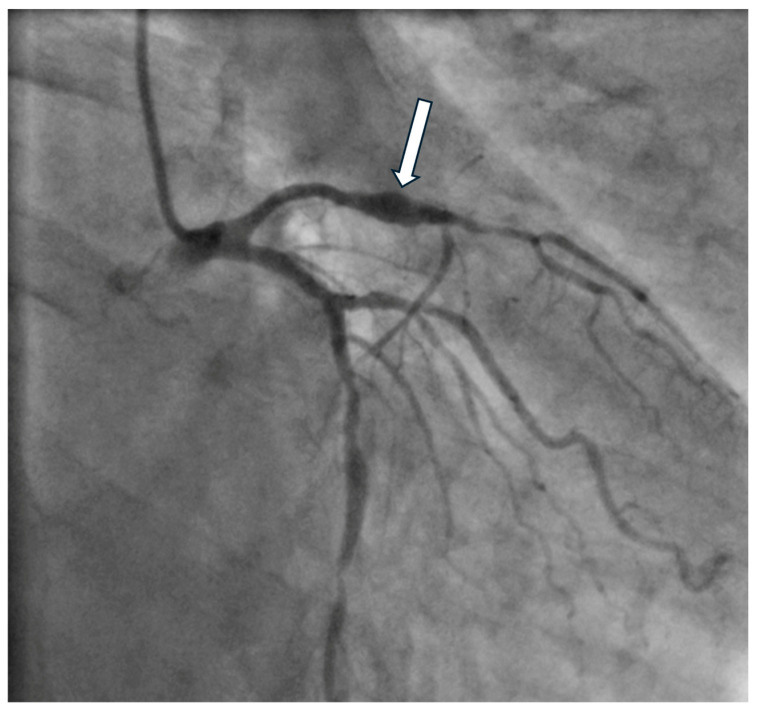
Coronary angiography of the fusiform aneurysm (arrow) of the left anterior descending artery.

**Figure 6:**
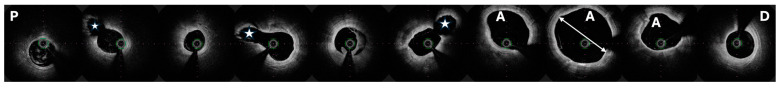
OCT of the left anterior descending artery before the aneurysmal lesion, within and after the aneurysmal lesion. We have given many cross-sections to present the proximal and distal vessel references and aneurysm-to-reference ratio, showing how large the lesion is. We have also provided the legend to make it easily readable: A—aneurysm; P—proximal; D—distal; arrows—maximal vessel diameter of aneurysm; asterisk—side branch.

Owing to the significantly higher resolution of imaging, optical coherence tomography (OCT) allows for a detailed assessment of the coronary vessel, including the morphology of atherosclerotic plaques and aneurysm complications, i.e., the presence of a thrombus (Figure 5 and Figure 6). However, the limitation is the limited penetration depth. In the case of larger aneurysms, it is impossible to visualize the entire cross-section of the vessel [54].

**Figure 7:**
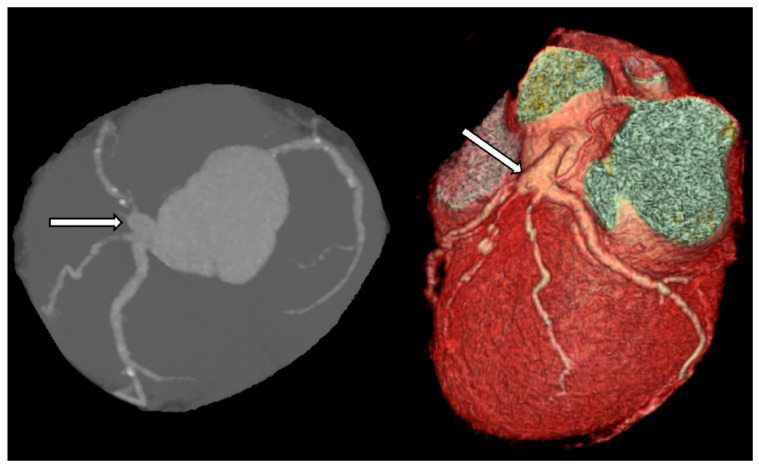
The aneurysmal lesion (arrow) of the left main coronary artery in CT.

Coronary computed tomography (CT) is a noninvasive method that enables accurate assessment of aneurysm size and location. It also allows for the evaluation of the amount of thrombus and calcification within the aneurysm (Figure 7). Multidetector computed tomography (MDCT) is particularly helpful in preoperative planning, as it depicts spatial relations of the aneurysm and other structures located nearby [55,56].

## 5. Clinical Significance

Although patients with CAAE may not present any clinical manifestations [9], they usually present classic symptoms of ischemic heart disease. They are associated with myocardial ischemia caused by micro-embolisms originating from the lumen of the aneurysm and leading to the closure of the distal segments of the vessel lumen, blood flow disorders, vascular spasm, and coronary microvascular dysfunction (CMD) [57]. Papadakis et al. found that CAE was associated with higher TIMI frame count values, indicating a significantly slow coronary flow [58]. The authors also found that the occurrence of CAE was associated with a deterioration of myocardial perfusion (myocardial blush grade) and a decrease in the coronary flow reserve [59,60]. 

CMD is the most probable cause of malperfusion of the myocardium in patients with no CAD. The pathophysiological factors include functional (systolic and diastolic) and structural (vascular remodeling) dysfunctions [57]. Iwańczyk et al. demonstrated that coronary microcirculation resistance, represented by the IMR value, was significantly higher in the CAAE group compared to non-CAAE patients with ischemia and no obstructive coronary arteries (INOCA). Adverse vascular remodeling is considered to be a possible mechanism of CAAE’s development [34,61]. Therefore, if the processes mentioned above involve the entire vascular bed, coronary microcirculation remodeling is thought to be a possible cause of CMD in patients with CAAE. Moreover, turbulent blood flow in aneurysms fosters the development of thrombi, which may result in distal embolization and, thus, impair coronary microcirculation [62].

Nevertheless, the coexistence of CAAE and significant stenosis of coronary arteries is very common (> 50%). In such cases, it is impossible to distinguish the mechanism of ischemia caused by CAAE from that of ischemia caused by atherosclerotic plaques that critically narrow the lumen of the artery. Another symptom that can be associated with CAAE is dysphagia, which is a consequence of the pressure of a giant aneurysm on the esophagus [63]. Symptoms of congestive right heart failure may also occur due to compression and obstruction of blood flow to the right atrium. 

The International Coronary Artery Aneurysm Registry (CAAR), published in 2017, was the first piece of research to assess the therapeutic management and clinical outcomes of patients with coronary aneurysms [18]. The study included 1565 eligible patients in the CAAR for final analysis. The CAAR recruited patients from 32 hospitals across nine countries (Canada, Cuba, the Czech Republic, Germany, Italy, the Netherlands, Spain, the United States, and Uruguay) [9]. Compared with the male patients, the female patients (*n* = 336; 21% of the total cohort) were older at presentation and more often hypertensive. Women less frequently had a smoking habit, had previous CAD, had a history positive for aortic aneurysms or related pathology, and less frequently had severe coronary stenosis. They were more often treated conservatively compared to men and received significantly less frequently aspirin or dual antiplatelet therapy (DAPT) at discharge. The duration of the median DAPT was also shorter (3 vs. 9 months, *p* = 0.001). Nonetheless, Kaplan–Meier analysis revealed no sex differences in death, MACEs, or bleeding during a median 37-month follow-up duration, although men more often experienced acute coronary syndrome (ACS) during follow-up (15% vs. 10%, *p* = 0.015).

In another study, the team analyzed sex disparities in treatment strategy in patients with CAA [64]. The results showed a comparable high-risk cardiovascular risk profile for both genders. Conservative treatment was more favorably implemented in female patients. Women received DAPT less often at discharge, with a shorter DAPT duration. Acute coronary syndrome (ACS) was more often diagnosed in male patients. Nevertheless, there were no significant differences in the overall clinical outcomes of male and female patients during follow-up [64].

CARED-POL is another multicenter observational nationwide registry of CAAE, conducted in cooperation with the Scientific Platform of the Polish Society of Cardiology (NCT06057987) [16]. The authors’ scientific interest and intensive work with patients with CAAE led to the creation of a registry that aims to thoroughly investigate the prevalence, morphology, risk factors for the development and complications of CAAE, and long-term prognosis in the Polish population. The registry was a continuation of the authors’ work on patients with CAAE. Hence, at the beginning, it was a national registry, and in the next step it was a prospective international registry. The registry will gather crucial information for selecting patients for further analysis of genetic factors. One of the goals is the subsequent genetic analysis. 

Another registry, the International Kawasaki Disease Registry, analyzed medium-term complications in patients with CAA after Kawasaki disease. The authors concluded that the medium-term risk of complications is established for those with maximum CAA Z-scores ≥ 10 (CAA is graded using Z-score systems). However, the problem still requires further risk stratification and close follow-up, including advanced imaging [65].

## 6. Complications of CAAE

**Figure 8:**
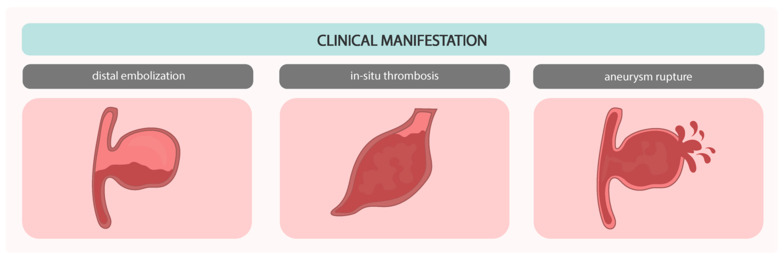
Illustration of the clinical manifestations of CAAE.

Complications of CAAE include acute myocardial infarction caused by embolism, vasoconstriction, malignant arrhythmias, and spontaneous artery dissection, as well as rupture of aneurysm resulting in cardiac tamponade or fistula to one of the heart cavities, the aorta, or the pulmonary arteries [14]. For this reason, the approach to each patient with CAAE should depend on the severity of symptoms, anatomical structure, size, and location of the aneurysm. However, the most common complications are coronary spasm, local thrombosis, distal embolization, coronary artery rupture, and compression of adjacent structures by giant coronary aneurysms (Figure 8) [66,67].

## 7. Prognosis

In the CASS study, the authors observed no differences in 5-year survival between patients with aneurysms and patients without aneurysms but with coronary artery disease. Therefore, the authors suggested that this anomaly can be considered a form of coronary artery disease [1]. Alternatively, Baman et al. found that the 5-year survival rate of patients with CAAE was only 71%, and they therefore recommended aggressive treatment in this group of patients [7]. MACEs occur in up to 10% of CAAE patients annually [9]. For example, in-aneurysm thrombosis leading to arterial occlusion or distal embolization can lead to MI [68,69,70]. To conclude, CAAEs are associated with both increased risk of MI and recurrence of MI.

## 8. Treatment

Given the unclear prognosis of CAAE, treatment should be tailored to each specific patient to avoid possible (sometimes even fatal) complications of CAAE. Patients can be offered conservative or invasive treatment.

### 8.1. Conservative Treatment

Conservative treatment depends on the etiology of CAA. If the causative factor is atherosclerosis, physicians should optimize the treatment of atherosclerosis, perform ischemic heart disease control, and alleviate the impact of cardiovascular risk factors.

In patients with incidentally discovered CAAE, the role of dual antiplatelet therapy (DAPT) or therapeutic anticoagulation remains unclear. Patients with concomitant CAD are treated according to the guidelines. However, we still lack sufficient high-quality evidence to unanimously justify the administration of DAPT or anticoagulants in patients with CAAE but without CAD. The conclusions of published studies are varied. Some retrospective studies indicated that differences in adverse event rates in patients with and without CAE did not have prognostic relevance. Hence, different management methods were not suggested [4,71,72]. 

Nevertheless, the authors of other studies drew alternative conclusions. Baman et al. disclosed how, in the enrolled patients, the incidence of an aneurysm had an independent adverse effect on long-term mortality rates. Hence, the belief that all patients with angiographic evidence of coronary aneurysms should receive aggressive modification of cardiovascular risk factors, regardless of the presence of CAD [7].

In another study, Warisava et al. showed that patients with coronary aneurysms had a higher rate of MACEs (54%) at 49-month follow-up [73]. The authors considered that the prognosis of CAA was directly related to the severity of the concomitant obstructive CAD [1,2,5]. Nevertheless, nearly one-third of MACEs in their study were associated with non-coronary vascular disease.

Another study by Doi et al. investigated 1698 patients with acute MI [74]. They compared the occurrence of MACEs in patients with and without CAE. The authors suggested a possible advantage of anticoagulation in patients with CAE and acute coronary syndrome. They concluded that CAE was associated with higher rates of MACEs at 49-month follow-up. However, regardless of the higher risk of CAE-related cardiac events, CAE patients treated with oral anticoagulation and who achieved a time in the target therapeutic range >60% had no occurrence of MACEs, compared with patients with time in the target therapeutic range <60% or without anticoagulation therapy (*p* = 0.03).

Nonetheless, it should be emphasized that the studies mentioned above have several crucial limitations, mainly due to their retrospective nature, small sample size, and other potential biases. 

Patients diagnosed with microcirculation dysfunction are treated according to the guidelines prepared and published by the European Society of Cardiology Working Group on Coronary Pathophysiology and Microcirculation [75]. Calcium channel blockers are recommended if vasospastic angina is confirmed in a vasoreactivity test [75].

The voices on the role of angiotensin-converting enzyme inhibitors (ACEIs) in preventing or slowing the progression of coronary aneurysms are not unanimous. Due to the role of the relationship between the ACE gene polymorphism and CAE, the administration of ACEIs also seems to be justified [27]. However, this still requires further investigation.

Statins may also be helpful due to their effect on inhibiting MMPs [76,77,78]. The study by Izidoro-Toledo et al. showed differences in the effects of statins on MMPs’ activities [78]. More lipophilic statins (simvastatin and atorvastatin) downregulate MMP-9 activity (but not the hydrophilic statin pravastatin). However, atorvastatin and pravastatin can downregulate the activity of MMP-2. 

Administration of vasodilators such as nitrates may impair coronary blood flow and exacerbate myocardial ischemia in patients with CAAE. Hence, it should not be implemented [79]. 

There is also limited evidence that anticoagulation may reduce thrombotic events in patients with Kawasaki disease. Therefore, the current guidelines recommend anticoagulation only in selected Kawasaki patients with large or rapidly expanding CAA [30].

In patients with Kawasaki disease, it has been shown that the administration of intravenous immunoglobulins (IVIGs) within ten days of the onset of symptoms reduces the incidence of coronary aneurysms from 23% to 5% and giant aneurysms from 5% to 1% [80]. The use of IVIGs in patients with Kawasaki disease can improve CAA regression and reduce the occurrence of MACE [81,82]. The data on invasive approaches, i.e., coronary angioplasty of aneurysmal arteries in pediatric patients and young adults with Kawasaki disease, are scarce [83,84]. These data reveal that the walls of a stenotic aneurysmal artery in Kawasaki patients may be accompanied by considerable thrombus. Therefore, IVUS is often necessary to assess the actual diameter of the vessel. The data also suggest that repeat intervention is common among Kawasaki patients following PCI. This is why the American Heart Association guidelines recommend restricting PCI treatment in Kawasaki patients with a single-vessel or focal multivessel disease [81].

### 8.2. Invasive Treatment

We still lack precise and unambiguous recommendations for the invasive management of patients with CAAE without ACS. There is a lack of data regarding the unfavorable course of the CAAE. Hence, the decision regarding each patient should be individually and thoroughly considered. 

Although the literature contains some descriptions of the effective invasive treatment of CAE with a stent, we should remember its possible consequences, especially the risk of in-stent restenosis (ISR) and thrombosis (prolonged epithelialization within the stent graft). According to the authors, this limits the use of stent grafts to the treatment of patients with concomitant significant coronary artery stenosis or giant coronary aneurysms, and to the treatment of CAE complications, e.g., acute coronary syndromes caused by the dissection of the vessel. The use of self-expanding stents coated with tetrafluoroethylene (PTFE) can be considered in giant aneurysms in tortuous arteries, but data on this subject are scarce [85,86]. 

Percutaneous treatment of CAA is associated with some challenges. The market does not offer stents that would be specifically designated for the treatment of CAA. Nonetheless, some stent graft systems have been used off-label to exclude CAA.

However, an intervention must be considered in some patients with CAA manifesting high-risk clinical or anatomical features. The treatment options should be adjusted individually according to the shape and size of the aneurysm. For instance, saccular aneurysms and small pseudoaneurysms can be treated with covered stent implantation unless involving a major side branch. Saccular or fusiform aneurysms involving a major side branch can be treated with balloon- or stent-assisted coil embolization or with surgical exclusion [62].

Surgical approaches and resection should be considered as a first-line therapy in case of CAA involving the left main coronary artery, multiple or giant (>20 mm, or >4 × reference vessel diameter) CAAs, and for saphenous vein graft aneurysms (SVGAs). 

Nevertheless, percutaneous closure with Amplatzer occluders or coil embolization with or without PCI of the native vessel can be an alternative to surgical treatment in symptomatic patients with a rapidly expanding CAA or large CAA causing external compression [62,87].

Data are available on the use of rheolytic thrombectomy in managing aneurysmal or ectatic infarct-related vessels. The AngioJet device (Boston Scientific, Marlborough, Massachusetts) demonstrated effectiveness in two randomized trials by decreasing embolization in patients who underwent PCI on venous grafts or native coronary vessels with massive thrombosis [88]. A few case reports have also proven its efficacy in patients with CAA [89,90].

Implementation of covered stents can also be problematic. Covered stent placement can be complicated and challenging due to severe tortuosity, calcification, or risk of side branch compromise. In such cases, it is possible to use the stent-assisted or balloon-assisted coil embolization (followed by adjunctive stent deployment across the aneurysm neck), which is a widely used technique in the treatment of cerebral aneurysms [91,92].

One can consider surgical treatment in patients with multiple aneurysms with concomitant significant stenosis in the coronary arteries, as well as in the case of giant aneurysms of the left main coronary artery, aneurysms causing pressure symptoms, fistulas, and other life-threatening complications [14]. Some authors believe that, due to the very high risk of complications, an aneurysm with a diameter exceeding 3–4 times the diameter of the reference vessel lumen is an indication for surgical intervention [14]. The surgery involves extraction, ligation, and resection or marsupialization with interposition graft of the aneurysm, with subsequent vessel reconstruction and coronary artery bypass grafting (CABG), if necessary [93,94,95].

## 9. Conclusions

The phenomenon of coronary artery aneurysm or ectasia (CAAE) is not an uncommon finding in coronary angiography. Despite the available data, the exact pathogenesis is still not well established. Moreover, the data regarding the prognosis and treatment of patients with CAAE are not unanimous. Hence, there is still a need for further research in this field of medicine.

## Figures and Tables

**Table 1:** Classification of CAE according to Markis et al. [3].

Type I	Diffuse ectasia of two or three vessels
Type II	Diffuse disease in one vessel and localized disease in another vessel
Type III	Diffuse ectasia of one vessel only
Type IV	Localized or segmental ectasia

**Table 2:** Classification of vessel wall structure in patients with CAAE by IVUS.

True Aneurysm	Preserved Vessel Wall Integrity within Three Layers (Intima, Media, and Adventitia)
Pseudoaneurysm	Loss of vessel wall integrity and damage to the adventitia
Complex plaque with aneurysmal appearance	Stenoses with ruptured plaques or spontaneous or unhealed dissections
Normal segment with aneurysmal appearance	Normal segment adjacent to stenosis

## Data Availability

No new data were created or analyzed in this study. Data sharing is not applicable to this article.
